# Commensal *E. coli* Stx2 lysogens produce high levels of phages after spontaneous prophage induction

**DOI:** 10.3389/fcimb.2015.00005

**Published:** 2015-02-03

**Authors:** Hildegunn Iversen, Trine M. L' Abée-Lund, Marina Aspholm, Lotte P. S. Arnesen, Toril Lindbäck

**Affiliations:** Department of Food Safety and Infection Biology, Norwegian University of Life SciencesOslo, Norway

**Keywords:** EHEC, Stx2, bacteriophage lambda, lysogen, commensal *E. coli*

## Abstract

Enterohemorrhagic *E. coli* (EHEC) is a food-borne pathogen that causes disease ranging from uncomplicated diarrhea to life-threatening hemolytic uremic syndrome (HUS) and nervous system complications. Shiga toxin 2 (Stx2) is the major virulence factor of EHEC and is critical for development of HUS. The genes encoding Stx2 are carried by lambdoid bacteriophages and the toxin production is tightly linked to the production of phages during lytic cycle. It has previously been suggested that commensal *E. coli* could amplify the production of Stx2-phages and contribute to the severity of disease. In this study we examined the susceptibility of commensal *E. coli* strains to the Stx2-converting phage ϕ734, isolated from a highly virulent EHEC O103:H25 (NIPH-11060424). Among 38 commensal *E. coli* strains from healthy children below 5 years, 15 were lysogenized by the ϕ734 phage, whereas lytic infection was not observed. Three of the commensal *E. coli* ϕ734 lysogens were tested for stability, and appeared stable and retained the phage for at least 10 cultural passages. When induced to enter lytic cycle by H_2_O_2_ treatment, 8 out of 13 commensal lysogens produced more ϕ734 phages than NIPH-11060424. Strikingly, five of them even spontaneously (non-induced) produced higher levels of phage than the H_2_O_2_ induced NIPH-11060424. An especially high frequency of HUS (60%) was seen among children infected by NIPH-11060424 during the outbreak in 2006. Based on our findings, a high Stx2 production by commensal *E. coli* lysogens cannot be ruled out as a contributor to the high frequency of HUS during this outbreak.

## Introduction

Enterohemorrhagic *Escherichia coli* (EHEC) causes disease with manifestations ranging from mild diarrhea to severe illness comprising hemorrhagic colitis (HC) (Riley et al., 1983) and hemolytic uremic syndrome (HUS) (Karmali et al., 1983, 1985). The *E. coli* serotype O157:H7, which caused the first described EHEC outbreak in 1982 (Riley et al., 1983) is so far the best described EHEC serotype. However, non-O157:H7 serotype strains have been implicated in a number of outbreaks and sporadic cases of HC and HUS (Luna-Gierke et al., 2014). In Europe, *E. coli* serotypes such as O103:H25 and O104:H4 have caused severe outbreaks (Schimmer et al., 2008; Beutin and Martin, 2012).

EHEC strains possess a range of colonization and virulence factors that facilitate infection and contribute to development of disease. Shiga toxin (Stx) is recognized as one of the main virulence factors in enterohemorrhagic disease caused by *E. coli* and all EHEC strains produce one or both of the Shiga toxins Stx1 and Stx2 (Scotland et al., 1985). Stx2 has been shown to be far more potent (as quantified by LD_50_ in mice) than Stx1 and patients infected with the latter are much less likely to develop serious illness than those infected by the former (Tesh et al., 1993; Friedrich et al., 2002). After being produced in the large intestine the Stx toxin passes through the epithelial cells and is disseminated via the blood stream to the target organs. Stx binds specifically and with high-affinity to the glycosphingolipid receptor globotriaosylceramide (Gb3) which is highly expressed in kidney cells (Jacewicz et al., 1986; Hughes et al., 2000; Okuda et al., 2006; Shimizu et al., 2007; Shin et al., 2009). After binding to the receptor, Stx is translocated into the cytosol where it causes cell damage by inhibiting protein synthesis (Sandvig and van Deurs, 2000). The Stx induced cell damage appears to be central in the pathogenic events leading to HUS and occasionally chronic kidney disease (Obrig, 2010; Obrig and Karpman, 2012).

About 15 years ago, several *E. coli* and *Shigella* strains were lysogenized with labeled Stx2 phages *in vitro* (Schmidt et al., 1999; James et al., 2001), and successful *in vivo* transduction experiments with Stx derivative phages have also been reported (Acheson et al., 1998; Toth et al., 2003). When a bacterial cell is infected by an Stx-encoding phage, two different pathways are possible (Allison, 2007). During lytic infection, the phage DNA exists as a separate molecule within the cell and utilizes the host machinery to express its genes and to produce large amounts of new phage particles until the host cell bursts. The other outcome is lysogenic infection, where the phage genome is integrated into the chromosome as a prophage, and is replicated along with the host genome. The phage can remain in the lysogenic state as long as the phage genes are repressed. It has been shown in several studies that the *stx2* genes are controlled by the phage late gene promoter, and that phage production is tightly linked to production of Stx toxin (Neely and Friedman, 1998; Unkmeir and Schmidt, 2000; Zhang et al., 2000; Wagner et al., 2002). Upon induction, the prophage can switch from the lysogenic state to the lytic cycle, accompanied by production of Stx and new phage particles (Herold et al., 2004; Waldor and Friedman, 2005). Several physical and chemical agents may act as prophage-inducing agents and all share the ability to activate the bacterial SOS response, mainly due to DNA damage (Kimmitt et al., 2000; Erill et al., 2007). Mitomycin C has often been used as prophage-inducing agent in studies of EHEC, however, H_2_O_2_ has been shown to be an effective prophage-inducer (Loś et al., 2009, 2010) and its presence in the gut may also increase Stx production (Wagner et al., 2001).

It has been reported that phages present in the gastrointestinal tract tend to enter the lysogenic pathway more often than the lytic pathway (Reyes et al., 2012). Factors like the number of infecting phages per bacterial cell and cell size prior to infection have been shown to influence whether the host will lyse or become lysogenic (St-Pierre and Endy, 2008). However, the mechanisms that determine the cell fate following phage-infection are complex and not fully understood.

Previous studies have shown that Stx-phages display a diverse host range, and also infect commensal *E. coli* (Wagner et al., 1999; Muniesa et al., 2003; Gamage et al., 2004). Gamage et al. (2004) demonstrated that commensal *E. coli* infected with Stx2 phages from *E. coli* O157:H7 were able to produce Stx2 and possibly increase the pathogenic potential of EHEC during infection. The contribution of commensal *E. coli* flora to Stx production was also demonstrated in a mouse model infected with *E. coli* O157:H7, where Stx was more commonly detected in mice colonized with *E. coli* sensitive to the Stx-phage than mice colonized with *E. coli* resistant to the Stx phage (Gamage et al., 2006). Children are particularly susceptible to EHEC infections and development of HUS (Tarr et al., 2005; Gyles, 2007). In 2006, Norway experienced a foodborne EHEC outbreak comprising 17 cases where all patients, except one (an adult aged 18), were children. The outbreak had an HUS frequency of 60%, which is extremely high, and all HUS patients were less than 9 years old (Schimmer et al., 2008). Due to the high HUS frequency, the causative strain, *E.coli* O103:H25 (NIPH-11060424), was considered to be particularly virulent (Schimmer et al., 2008). The strain was later shown to be closely related to the *E. coli* O104:H4 strain causing a large outbreak in Germany in 2011 (L' Abée-Lund et al., 2012). However, the genetic and phenotypic features underlying the extraordinary high virulence of the Norwegian outbreak strain are not yet known.

In this study, we examine the susceptibility of commensal *E. coli* isolates from young children to the Stx2-converting phage (ϕ734) from the 2006 Norwegian outbreak strain. We address the commensal *E. coli* strains sensitivity for lytic and lysogen infection and their ability to contribute to ϕ734 phage production and thereby Stx2 production.

## Materials and methods

### Bacterial strains and phages

The bacterial strains used in this study are listed in Table 1 and Supplementary Table 1. The commensal *E. coli* strains were isolated from fecal samples from healthy Norwegian children below 5 years of age in the years 2009–2014. All strains tested negative against Test Serum Anti-Coli O 103:K- and Anti-Coli O157:K- in agglutination tests (SIFIN, Germany). EHEC O103:H25 NIPH-11060424 is a highly virulent strain which caused a severe outbreak in Norway in 2006 (Schimmer et al., 2008; L' Abée-Lund et al., 2012). The phage infection experiments in this study were performed using the Stx2-converting phage ϕ734 from NIPH-11060424 (L' Abée-Lund et al., 2012) or the recombinant version of this phage (Table 1). The recombinant phage ϕ734 Cm in which *stx2A* is replaced by the chloramphenicol resistance gene (*cat*) was constructed by Dr. Muniesa, University of Barcelona, Spain, as described by Serra-Moreno et al. (2006). *E. coli* DH5α was used as a propagating strain for determination of phage concentration. A stable lysogen of the laboratory strain *E. coli* C600 carrying ϕ734 (C600:ϕ734) was created by infecting *E. coli* C600 with ϕ734. The lysogen was identified by PCR using the *stx2* primers listed in Table 2.

**Table 1:** ***E. coli* strains and bacteriophages used in the study**.

**Bacterial strains and phage**	**Characteristics**	**References**
***E. coli* LABORATORY STRAINS**		
C600	K-12 derivate	Appleyard, 1954
DH5α	K-12 derivate	Hanahan, 1985
**EHEC STRAINS**		
NIPH-11060424	Human isolate, Norwegian outbreak strain 2006, O103:H25. Possesses the Stx2-phage ϕ734 and the phi-like phage TL-2011b	Schimmer et al., 2008; L' Abée-Lund et al., 2012
**COMMENSAL *E. coli* ISOLATES**		
NVH-1034-NVH-1042, NVH-1064-NVH-1094	Child isolates (*n* = 38) non-O103/O157	This study
L1034-L1042, L1064-L1094	ϕ734 Cm lysogens of the commensal *E. coli* isolates with corresponding numbering	
**RECOMBINANT *E. coli* STRAINS**		
NIPH-11060424:ϕ734 Cm	ϕ734 Cm lysogen in NIPH-11060424^a^	This study
C600:ϕ734 Cm	ϕ734 Cm lysogen in C600^a^	This study
C600:ϕ734	ϕ734 lysogen in C600^a^	This study
**BACTERIOPHAGES**		
ϕ734	Stx2-converting phage from NIPH-11060424 (GenBank acc no JQ011318.1) Synonyme name is TL-2011c	L' Abée-Lund et al., 2012
ϕ734 Cm	ϕ734Δ*stxA::cat*	This study

a *The strains were stable*.

**Table 2:** **PCR primers used in the study**.

**Primers**	**Sequence (5′-3′)**	**References**
*stx2* forward	GCGTTTTGACCATCTTCGT	Muniesa and Jofre, 1998
*stx2* reverse	ACAGGAGCAGTTTCAGACAG	Muniesa and Jofre, 1998
*cat* forward	GGGCGAAGAAGTTGTCCATA	This study
*cat* reverse	TACACCGTTTTCCATGAGCA	This study
phi-phage forward	GCGGTCATGAAAACAAACCT	This study
phi-phage reverse	AGGCGGCAGGATTTATCAAG	This study

### Preparation of phage filtrates for phage infection experiments

*E. coli* strains carrying either ϕ734 or ϕ734 Cm were grown in Lysogeny broth (LB) to mid-exponential growth phase (OD_600_ = 0.3–0.5) and then left non-induced or induced by addition of either Mitomycin C (MMC) (0.5 μg/ml) or H_2_O_2_ (1.5 mM). The cultures were then further incubated overnight at 37°C followed by centrifugation for 10 min at 4500 rpm and sterile-filtrated using 0.22 μm filters (Millex-GP, Millipore, Bedford, MA). The phage concentration in the bacteria-free filtrate was determined by plaque assay using *E. coli* DH5α as a propagating strain. In order to remove any colicins, Trypsin (Sigma) was added to the phage-filtrate to a final concentration of 0.1 mg/ml followed by 1 h incubation at 37°C (Gordon and O'Brien, 2006).

### Plaque assay

A plaque assay was used to determine the concentration of infective phage particles in the phage filtrates. A volume of 100 μl of phage filtrate was mixed with 900 μl of *E. coli* DH5α culture (OD_600_ = 0.3) containing 10 mM CaCl_2_, and then further incubated without agitation for 30 min. After incubation, the samples were mixed with 2.5 ml 0.7% LB agar and poured onto LB agar plates containing 10 mM CaCl_2_. The plates were incubated overnight at 37°C and plaques were counted. The phage concentration is given as plaque forming units/ml (PFU/ml).

Hybridization of plaques in *E. coli* DH5α lawn was performed to confirm that *E. coli* DH5α was susceptible to ϕ734 and ϕ734 Cm (see the Materials and Methods below). The results showed that 100% of the plaques were positive for the corresponding probe, either *stx2A* or *cat*. Thus, *E. coli* DH5α was used to quantify the number of phages in the phage filtrates.

### Plaque hybridization

Plaque hybridization was performed according to a standard procedure (Datz et al., 1996; Sambrook and Russell, 2001) using Hybond-N+ membranes (Amersham Pharmacia Biotech). The membranes were hybridized against a DIG labeled PCR amplified probe (primers shown in Table 2). Labeling of probe and hybridization were performed using the DIG-High Prime DNA Labeling and Detection Starter Kit I (Roche Diagnostics, Mannheim, Germany) according to the manufacturer's instruction. The hybridization temperature used for all experiments was 56°C.

### Lytic phage infection

To test for susceptibility to lytic infection, 5 μl of ϕ734 phage-filtrate was spotted on LB soft agar plates with 10 mM CaCl_2_, containing commensal *E. coli* strains, *E. coli* C600 or *E. coli* DH5α. The concentrations of the ϕ734 phage-filtrates used in the spot assay were 10^6^ PFU/ml when propagated on NIPH-11060424 and 10^9^ PFU/ml when propagated on *E. coli* C600. The LB plates were incubated overnight at 37°C. The susceptibility to lytic infection among the commensal *E. coli* strains were additionally tested using the plaque assay where the recipient culture had a cell density of 1 × 10^8^ CFU/ml (OD_600_ = 0.3) and the ϕ734 phage concentration was either 1 × 10^8^ PFU/ml or 1 × 10^5^ PFU/ml, giving a multiplicity of infection (MOI) of 1 and 0.001, respectively.

### Lysogen infection

The recombinant ϕ734 Cm phage was used to test commensal *E. coli* strains for susceptibility to lysogenic infection as described previously (Schmidt et al., 1999). The commensal *E. coli* strains were tested for Cm sensitivity prior to the experiment, and all strains were found sensitive. Colonies growing on LB plates containing 25 μg/ml of chloramphenicol were considered to be lysogens. Lysogens from each commensal strain were named by adding the prefix L to their wildtype number. Phage filtrate from 13 lysogens (Table 3) was prepared to examine their phage production by plaque assay using DH5α as a recipient strain. The stability of the ϕ734 Cm phage containing lysogens was tested by culturing lysogens in LB without antibiotic selection for 10 passages. After each passage, dilutions of the cultures were spread onto LB plates with chloramphenicol to examining the level of bacteria carrying ϕ734 Cm.

**Table 3:** **Susceptibility of 38 commensal *E. coli* strains to lysogenic infection by the ϕ734 Cm phage**.

***E. coli* isolates**	**ϕ734 Cm from NIPH-11060424: ϕ734 Cm MOI 0.005**	**ϕ734 Cm from C600: ϕ734 Cm MOI 0.005**	**ϕ734 Cm from C600: ϕ734 Cm MOI 0.5^a^**	**ϕ734 Cm from NVH-1090: ϕ734 Cm MOI 0.5**
NVH-1034	−/−	−/−	−/−	−/−
NVH-1036	−/−	−/−	−/−	−/−
NVH-1037	−/−	−/−	40/20	30/50
NVH-1038	−/−	−/−	−/−	−/−
NVH-1039	−/−	−/−	−/−	−/−
NVH-1040	−/−	−/−	−/−	−/−
NVH-1041	−/−	−/−	−/−	−/−
NVH-1042	−/−	−/−	−/−	−/−
NVH-1064	−/−	200/10	10/90	300/200
NVH-1065	−/−	−/10	200/600	20/1000
NVH-1066	−/−	20/−	200/30	30/20
NVG-1067	−/−	−/−	200/10	40/100
NVH-1068	−/−	−/−	−/−	−/−
NVH-1069	−/−	−/−	−/−	−/−
NVH-1070	−/−	−/−	−/−	−/−
NVH-1071	−/−	−/−	−/−	−/−
NVH-1072	−/−	−/−	−/−	−/−
NVH-1073	−/−	−/−	−/−	−/−
NVH-1074	−/−	−/−	−/−	−/−
NVH-1075	−/−	−/−	−/−	500/20
NVH-1076	−/−	−/−	−/−	−/−
NVH-1077	30/100	−/−	1200/400	30/700
NVH-1078	2000/100	500/2000	10000/8000	3000/8000
NVH-1079	−/−	−/−	−/−	−/−
NVH-1080	−/−	−/−	−/−	−/−
NVH-1081	−/−	−/−	10/50	30/20
NVH-1083	−/−	−/−	−/−	−/−
NVH-1084	−/−	−/−	30/30	−/−
NVH-1085	−/−	−/−	−/−	30/200
NVH-1086	−/−	70/−	50/300	100/20
NVH-1087	−/−	−/−	−/−	−/−
NVH-1088	−/−	10000/50000	10000/10000	8000/9000
NVH-1089	−/−	−/−	−/−	−/−
NVH-1090	40/200	100/400	4000/3000	3000/2000
NVH-1091	−/−	−/−	−/−	−/−
NVH-1092	−/−	−/−	−/−	−/−
NVH-1093	−/−	−/−	60/40	30/80
NVH-1094	−/−	−/−	−/−	−

*The phage was propagated on three different strains (the original outbreak strain NIPH-11060424, the laboratory E. coli strain C600 and the commensal E. coli strain NVH1090), and two different concentrations of phages (MOI 0.005 and MOI 0.5) were used. The results are presented as the number of lysogens/ml. Two replicates were performed for all conditions*.

*−, no lysogens detected*.

a *lysogens made under this condition were selected for further examination of phage production (**Figure 2**)*.

### Semi-quantification of Stx2 levels by VTEC-RPLA kit

A VTEC RPLA-toxin detection kit (Oxoid Limited, Basingstoke, UK) was used to determine the Stx2 production by NIPH-11060424 and C600:ϕ734. The assay was performed according to the manufacturer's instruction. The amount of sample in each test well was reduced 2-fold at each dilution. The Stx2 titer was defined as the reciprocal of the highest dilution causing latex agglutination.

### Western blot

Proteins were separated by electrophoresis using the NuPAGE Novex Bis-Tris gel systems (Invitrogen) and SeeBlue Plus2 Pre-Stained Standard (Invitrogen) as molecular weight marker. After electrophoresis, the proteins were transferred to a PVDF membrane (Millipore) according to standard protocols (Harlow and Lane, 1988). Stx2 in culture supernatants was detected using monoclonal antibodies against Stx2 (STX2-11E10, TOXIN TECHNOLOGY, INC., Sarasota, FL) diluted 1:1000. Biotin-conjugated anti-mouse antibodies from goat (Amersham Biosciences) were used as secondary antibodies (1:3000). A complex of streptavidin (Bio-Rad) and biotinylated alkaline phosphatase (Bio-Rad) was used at a dilution of 1:3000 prior to development with nitro blue tetrazolium/5-bromo-4-chloro-3-indolyl phosphate (Bio-Rad).

### Statistical analysis

Student's *t*-test was used to determine significant differences between groups. A *P* ≤ 0.05 was considered significant.

## Results

### Susceptibility of commensal *E. coli* strains to lytic infection by the Stx2-converting phage ϕ734

Thirty-eight commensal *E. coli* strains were tested for susceptibility to lytic infection by ϕ734 or ϕ734 Cm propagated on either in EHEC NIPH-11060424 or on the laboratory strain *E. coli* C600. None of the commensal *E. coli* strains were susceptible to lytic infection by any of the two phages propagated on NIPH-11060424 or *E. coli* C600 at any of the tested concentrations. Previous studies have shown that NIPH-11060424 carries a phi-like phage (TL-2011b) in addition to the Stx2 phage (L' Abée-Lund et al., 2012). This phi-like phage is 53% identical to bacteriophage Φ V10, a temperate phage that specifically infects *E. coli* of serogroup O157:H7 (Perry et al., 2009). TL-2011b was shown by spot assay and following hybridization using a phi-phage specific probe to infect *E. coli* of serogroup O103:H25, while none of the commensal strains tested were susceptible for lytic infection by this phage (Supplementary Table 2). This indicates that phage TL-2011b is serotype specific.

### Susceptibility of commensal *E. coli* strains to lysogenic infection by the Stx2-converting phage ϕ734 Cm

A total of 15 out of 38 (39%) commensal *E. coli* isolates were susceptible to lysogenic infection by ϕ734 Cm (Table 3). The number of lysogenic cells recovered varied considerably, from 10 CFU/ml to 10^4^ CFU/ml, between the different isolates. Two of the tested isolates (*E. coli* NVH-1078 and *E. coli* NVH-1088) seemed particularly susceptible to the ϕ734 Cm phage. The bacterial host in which the phage was produced also influenced the lysogenicity, as 8% (3/38) of the commensal isolates were susceptible to lysogenic infection by ϕ734 Cm propagated on NIPH-11060424 while 18% (7/38) was susceptible to ϕ734 Cm propagated on C600 when the multiplicity of infection were the same (MOI of 0.005) (Table 3). Within isolates, the number of lysogens increased with increasing phage concentration. The number of strains susceptible to ϕ734 Cm propagated on C600 increased from 18 to 34% (13/38) when the MOI was increased from 0.005 to 0.5 (Table 3). When the commensal isolates were infected with ϕ734 Cm, propagated on the commensal lysogenic *E. coli* strain 1090 at an MOI of 0.5, the number of strains susceptible to lysogenic infection increased to 39% (15/38) (Table 3).

**Figure 1:**
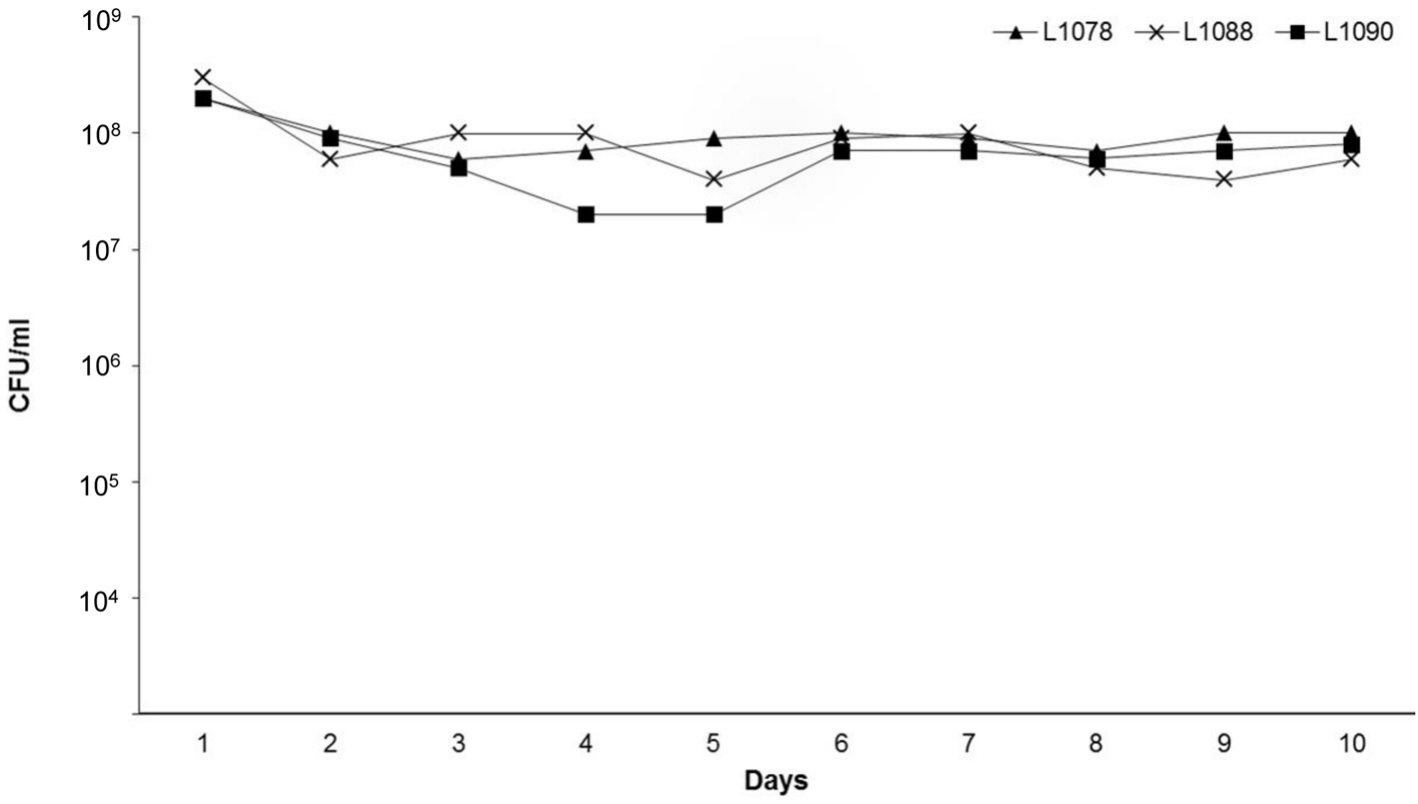
**Stability of the commensal *E. coli* lysogens L1078, L1088, and L1090 during 10 cultural passages in LB broth without chloramphenicol**. After each passage, the bacterial cultures were examined for loss of prophage by determining the number of Cm resistant colonies (CFU/ml).

The level of Cm resistant colonies remained constant during all cultural passages of the three lysogens (L1078, L1088, and L1090) that were tested for stability (Figure 1). This shows that ϕ734 Cm was stably maintained in the commensal hosts.

### Phage production by ϕ734 Cm lysogens under non-induced conditions and following treatment with MMC or H_2_O_2_

**Figure 2:**
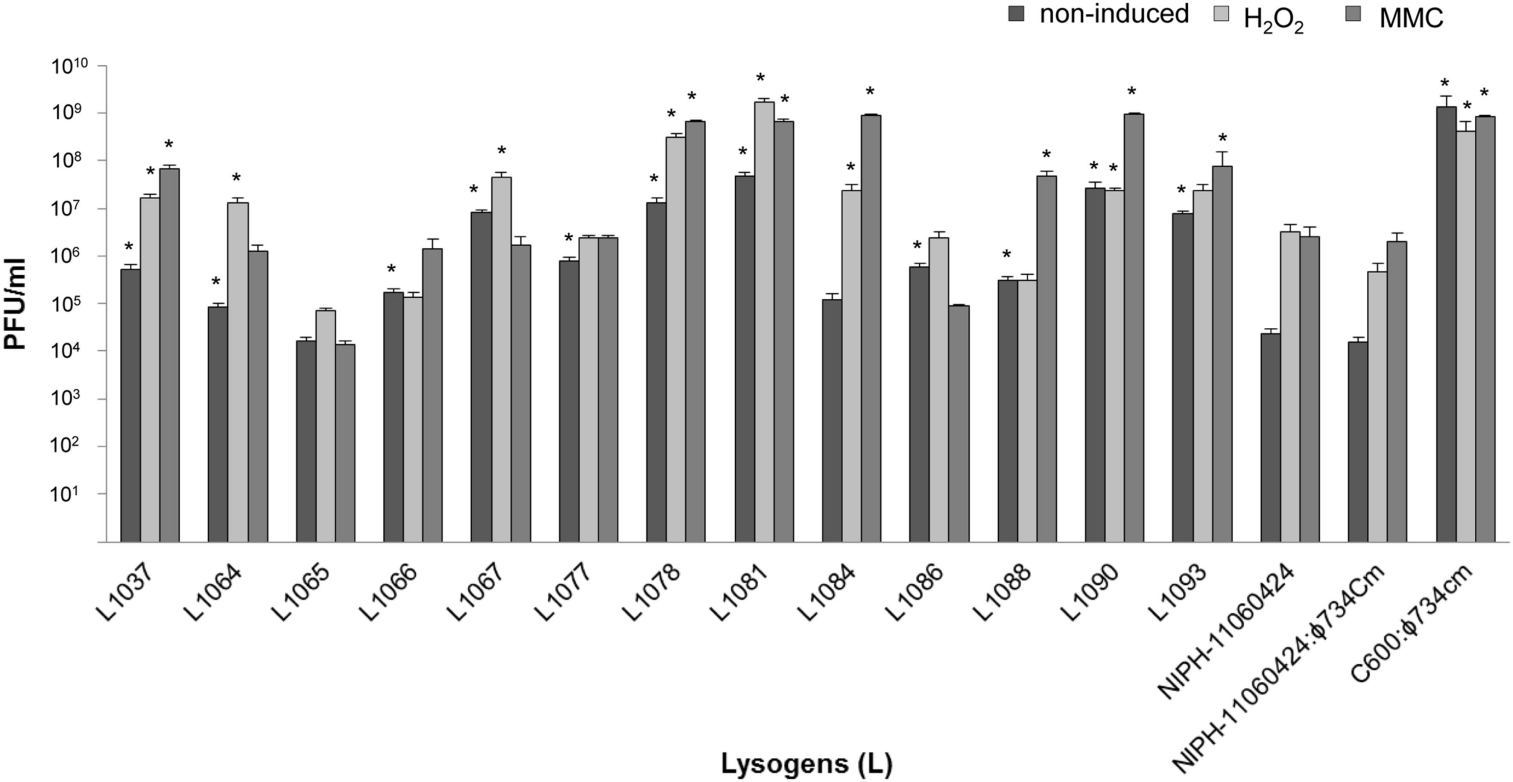
**Bar chart showing Stx2-phage production by NIPH-11060424, NIPH-11060424:ϕ734 Cm, C600:ϕ734 Cm and 13 commensal *E. coli* ϕ734 Cm lysogens under non-induced, MMC induced or H_2_O_2_ induced conditions**. The error bars represent the standard error of the mean (SEM) of three independent experiments. An asterisk indicates statistical significant difference (*P* < 0.05) in phage titer from lysogen compared to corresponding phage titer from NIPH-11060424.

The 13 commensal *E. coli* isolates that were susceptible to lysogenic infection by ϕ734 Cm propagated on *E. coli* C600 were selected for further studies (Table 3). These isolates were tested for phage production during spontaneous (non-induced) prophage induction and after induction with mitomycin C (MMC) or H_2_O_2_ (Figure 2). There was no difference in phage production between NIPH-11060424 carrying the original ϕ734 phage and NIPH-11060424 carrying ϕ734 Cm. Twelve out of 13 commensal lysogens (L1037, L1064, L1066, L1067, L1077, L1078, L1081, L1084, L1086, L1088, L1090, and L1093) produced significantly more phages than NIPH-11060424 under one or more of the tested conditions. The remaining commensal lysogen (L1065) produced less Stx2-phages compared to NIPH-11060424. The differences between non-induced and induced phage production (either by MMC or H_2_O_2_) were less than 2 log for all lysogens except L1084, which showed one of the highest MMC induced phage productions (10^9^ phages/ml). The non-induced culture of NIPH-11060424:ϕ734 Cm produced about 2 log less phages than the MMC or H_2_O_2_ induced cultures, which produced approximately equal numbers of phages. All the commensal *E. coli* lysogens produced more than 10^4^ phages/ml without induction, and L1081 and L1090 produced nearly as much as 10^8^ phages/ml in the non-induced cultures (Figure 2). Three lysogens (L1065, L 1067, and L1086) produced either equal amounts or more phages in the non-induced cultures than in the MMC induced cultures. These lysogens also showed 1–2 log greater phage production after induction with H_2_O_2_ than with MMC. Prior to the experiments, all the commensal *E. coli* strains were tested for the ability to produce phages after MMC induction by testing the culture filtrates in plaque assay (data not shown). Three of the commensal stains (NVH-1064, NVH-1077, and NVH-1086) carried MMC inducible phages naturally, of which none were of Stx type. The level of phage production in these strains was negligible (<10^3^ PFU/ml) compared to the phage production after ϕ734 Cm infection (>10^5^). Furthermore, the naturally carried phages formed larger plaques compared to the characteristic pin-point plaques formed by the Stx2-phages (results not shown) which made them easy to exclude when counting plaques formed by ϕ734.

### Phage production and Stx2 expression by *E. coli* C600:ϕ734

**Figure 3:**
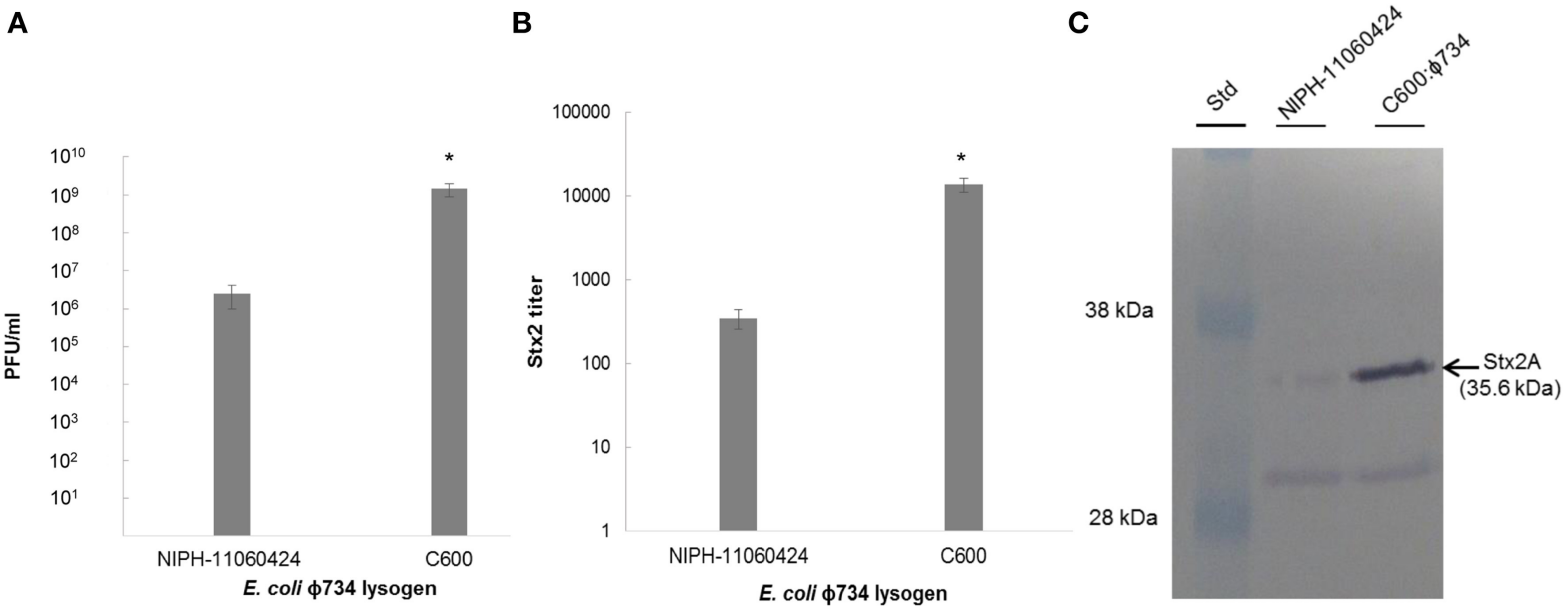
**Phage production and Stx2 expression by NIPH-11060424 and *E. coli* C600: ϕ734 after MMC induction. (A)** Phage production measured as plaque forming units. **(B)** Stx2 titer measured by reverse passive latex agglutination. **(C)** Stx2 production visualized by Western blot. The arrow indicates the Stx2A band. The error bars represent the standard error of the mean (SEM) of three independent experiments. An asterisk indicates statistical significant difference (*P* < 0.05) in phage production and Stx2 expression between C600:ϕ734 and NIPH-11060424.

While the production of phages was approximately 3 log higher in C600:ϕ734 than in NIPH-11060424 after MMC induction (Figure 3A), the Stx2 titer indicated that Stx2 production was 40 times higher in *E. coli* C600:ϕ734 than in NIPH-11060424 (Figure 3B). Western blot analysis of the phage filtrates confirmed the high Stx2 production by C600:ϕ734 (Figure 3C).

## Discussion

Children are usually more susceptible to EHEC infections and development of HUS than other groups. While some individuals exposed to the bacteria become ill others carry the bacteria asymptomatically, and the reason for this is still unknown. There is increasing evidence that commensal *E. coli* strains infected with Stx2-converting phages can contribute to Stx production in the intestine, and thereby increase the pathogenicity during EHEC infection (Gamage et al., 2003, 2004, 2006; Toth et al., 2003; Cornick et al., 2006). In this report, we provide results which suggest that some commensal *E. coli* have the potential to be significant producers of Stx and could have contributed to the extraordinary pathogenicity of strain NIPH-11060424 during the Norwegian 2006 EHEC outbreak.

We showed that 39% of commensal *E. coli* isolates from children were susceptible to lysogenic infection by a chloramphenicol resistant derivative of ϕ734. No lytic infection of the commensal *E. coli* isolates was observed which is consistent with the low rate of lytic infection by Stx2-encoding phages observed in other studies (Schmidt et al., 1999; James et al., 2001; Gamage et al., 2004; Reyes et al., 2012). The lysogenic infection rate observed here is comparable to the rates reported in other studies (Gamage et al., 2004). Gamage et al. (2004) found that 35% of *E. coli* isolates were susceptible to lysogenic infection by the Stx2-converting phage W933. The *E. coli* isolates tested in that study were of both clinical and non-clinical origin from animals and humans, and were therefore distinct from our study population. Recently, Tozzoli et al. (2014) showed that *E. coli* isolates representing the main *E. coli* pathogroups [enterotoxigenic *E. coli* (ETEC), enteropathogenic *E. coli* (EPEC), enteroinvasive *E. coli* (EIEC), enteroaggregative *E. coli* (EAggEC) and extraintestinal pathogenic *E. coli* (ExPEC)] were susceptible to infection by Stx2- phages. However, in contrast to the commensal *E. coli* isolates studied here, the pathogenic *E. coli* strains were only able to carry the Stx2-phages transiently (Tozzoli et al., 2014).

In accordance with other studies, we observed that an increased MOI resulted in an increased formation of lysogens (Zeng and Golding, 2011). However, we also observed that the strain used for ϕ734 Cm phage production influenced the susceptibility of the recipient strain to lysogenic infection. When ϕ734 Cm was produced in either *E. coli* strain C600 or L1090 it seemed to tolerate a broader host range compared to when it was produced in NIPH-11060424 (Table 3).

Phage production by strains NIPH-11060424 and NIPH-11060424:ϕ734 Cm was very similar under all tested conditions (Figure 2), indicating that replacing *stx2A* with the chloramphenicol resistance gene (*cat*) did not influence the behavior of the phage. The selective marker was convenient in the phage experiments, as it made retrieval of lysogens more feasible, but, the recombinant phage was of course unsuitable in experiments for Stx production. Unfortunately, due to the relatively low infection rate, we were not able to isolate a commensal *E.coli* strain lysogenized by the wild-type ϕ734 phage. However, we were able to retrieve the ϕ734 phage in *E. coli* C600 (C600:ϕ734). This lysogen enabled determination of Stx2 production in another genetic background than the original EHEC outbreak strain. Under the same conditions, *E. coli* C600:ϕ734 produced about 1000 times more Stx2-converting phage than the original EHEC outbreak strain, and about 40 times more Stx2 (Figure 3). Stx2 measurements could not be done in the commensal *E. coli* lysogens, however, based on the close linkage between phage production and toxin synthesis (Neely and Friedman, 1998; Unkmeir and Schmidt, 2000; Zhang et al., 2000; Wagner et al., 2002) we assume that the number of phage produced in these lysogens will mirror the amount of Stx2 that would have been produced by the native Stx2-converting phage. A similar discrepancy between increased phage-production compared to increased Stx2 production has been shown earlier by Zhang et al. (2000), where ciprofloxacin induction of an O157:H7 strain resulted a 1000 fold increase in phage production while the Stx2 production only increased 58 fold.

The laboratory strain *E. coli* C600 lysogenized with ϕ734 Cm produced as much as 10^9^ PFU/ml under non-induced conditions, which was the highest level of phage production observed during this study (Figure 2). Phage-production in the commensal *E. coli* ϕ734 Cm lysogens ranged from 10^4^ to nearly 10^8^ PFU/ml under both induced and non-induced conditions. This means that some commensal *E. coli* produced a considerably higher amount of Stx phage than NIPH-11060424, and also higher levels than EHEC O157:H7 EDL933, which produced about 10^6^ PFU/ml under identical non-induced conditions (Imamovic and Muniesa, 2012). The reason why different *E. coli* strains lysogenized by an identical phage, produce different amounts of phage is not known. However, the amount of phages produced is most probably dependent on the genetic background of the host strain e.g., the regulation of the SOS response and the phage repressor system in each strain will have an impact on phage production.

Since the Stx-prophage induction is closely linked to activation of the bacterial SOS-response and expression of host-encoded RecA protein (Fuchs et al., 1999; Kimmitt et al., 2000), the SOS-response inducing agent MMC is frequently used to activate the phage- and Stx production in EHEC (Fuchs et al., 1999; Schmidt et al., 1999; Muniesa et al., 2004). However, H_2_O_2_ may represent a more natural inducing agent, as it is produced in the gut as part of the innate immune response (Wagner et al., 2001). Five of the commensal *E. coli* ϕ734 Cm lysogens demonstrated higher phage production after H_2_O_2_ induction than after MMC induction. The levels of phage production in the non-commensal isolates NIPH-11060424, NIPH-11060424:ϕ734 Cm and C600:ϕ734 Cm were similar after H_2_O_2_ and MMC induction. The strong inducing capability by H_2_O_2_ seen in the commensal *E. coli* lysogens may have implications for disease, as H_2_O_2_ release occurs during *in vivo* EHEC infection (Wagner et al., 2001). Surprisingly, we also observed high production of phage in some of the lysogens under non-induced conditions. Five of the commensal *E. coli* ϕ734 Cm lysogens produced a higher amount of phage non-induced, than NIPH-11060424 did under either H_2_O_2_ or MMC induced conditions.

**Figure 4:**
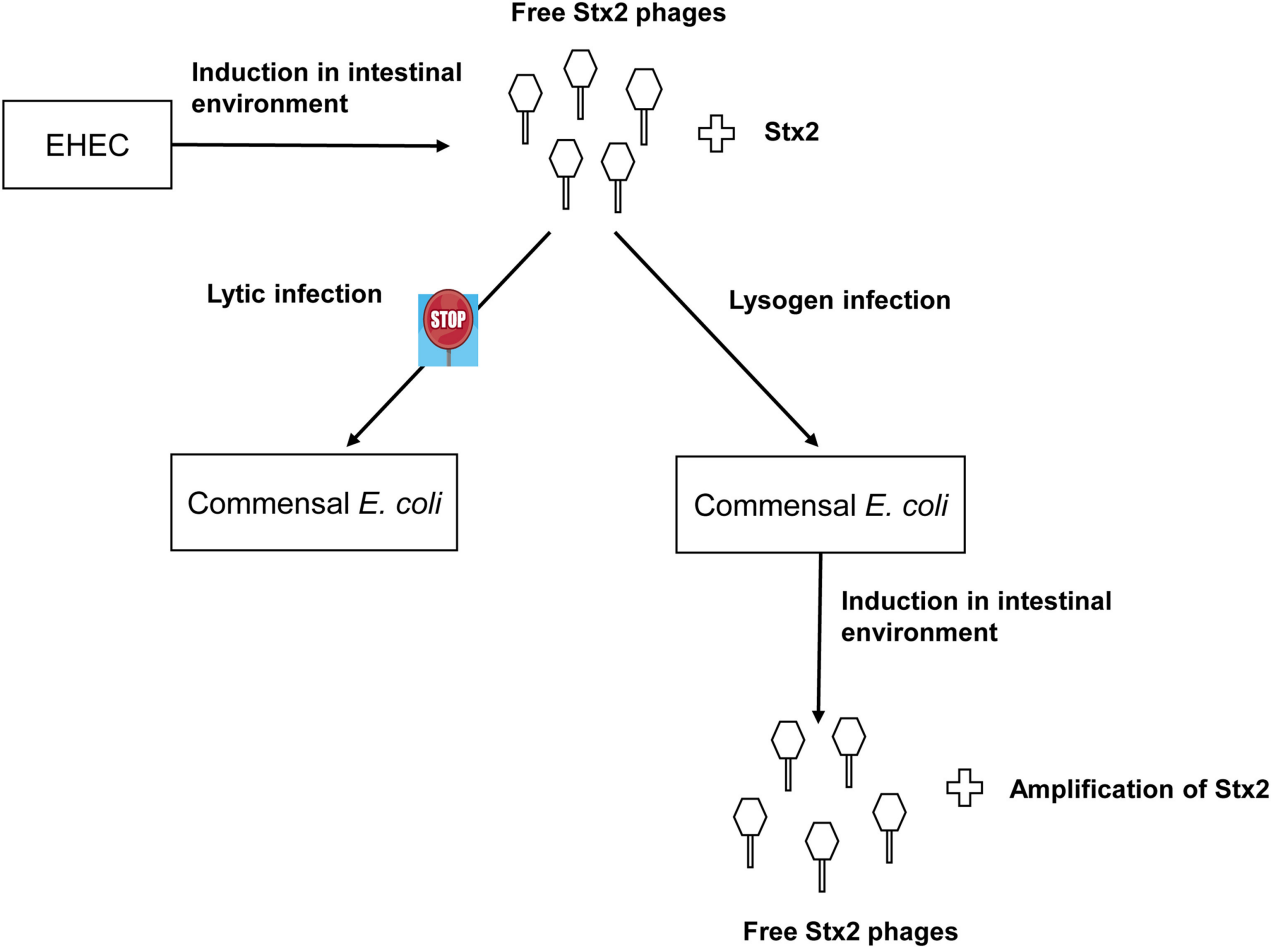
**Suggested model of commensal *E. coli* contribution to Stx2 production in the intestinal tract**. The Stx2-phage ϕ734 are produced by its EHEC host and infect susceptible commensal *E. coli* strains lysogenically. The commensal *E. coli* ϕ734 lysogens enter the lytic cycle either spontaneously or after exposure to inducing agents present in the intestinal environment. The commensal lysogens produce phages at a high frequency leading to a concomitant increase in Stx2 production.

The observed lack of lytic infection by the ϕ734 phage in the commensal *E. coli* isolates contrasts the high level of non-induced phage production in the corresponding lysogens. However, together these results indicate that commensal *E. coli* strains might contribute to Stx2 production through first becoming lysogenized and then subsequently enter the lytic cycle at a high frequency (Figure 4). It has previously been reported that spontaneous induction occurs more readily in Stx-phages than in other lambdoid phages (Livny and Friedman, 2004; Aertsen et al., 2005; Shimizu et al., 2009). However, spontaneous induction to the extent observed in this study has, to our knowledge, not previously been reported.

Since efficient Stx production only occurs after prophage induction followed by lysis and death of the host cell, one may expect that EHEC carrying these phages will eventually die out. Recently, Loś et al. suggested that prophages are induced at a low frequency in the gut which does not compromise the persistence of the EHEC population (Loś et al., 2012). There are various repressor systems that interfere with phage production in strains carrying several prophages (Burz et al., 1994; Serra-Moreno et al., 2008). The lower production of phages by NIPH-11060424 compared to strain C600:ϕ734 and several of the commensal *E. coli* lysogens may result from the presence of repressor systems originating from other prophages in the genome of NIPH-11060424. These repressor systems may act to keep a balance between the lysogenic and lytic infection and thereby benefit the survival of the EHEC population.

In conclusion, we observed that a high proportion of commensal *E. coli* is susceptible to infection by ϕ734 Cm and that some isolates were infected at a higher frequency than others. The ϕ734 Cm phage infected the commensal *E. coli* isolates only via the lysogenic pathway. Some of the commensal *E. coli* lysogens produced considerably higher amounts of phage particles than EHEC NIPH-11060424. These lysogens would also likely have produced high levels of Stx2 if they were lysogenized with the original Stx2-converting phage ϕ734 as modeled in Figure 4. This study supports the hypothesis that Stx2-converting phages are able to infect commensal *E. coli* strains, and thereby enhance Stx2 production during EHEC infection. Together our data strongly endorse that Stx2-converting phages released from EHEC in the gut can lysogenize commensal *E. coli* and turn them into effective Stx producers and thus enhance the pathogenicity of the EHEC infection. Therefore, it would be interesting to examine commensal *E. coli* isolates from asymptomatic EHEC carriers and from EHEC triggered HUS patients for Stx phage susceptibility and for the presence of lysogenic Stx-phages.

## Author contributions

All authors contributed to the design of the study, and to interpretation and analyses of the data. Hildegunn Iversen did the experiments and drafted the manuscript. Toril Lindbäck assisted in the experiments and in drafting the manuscript. Trine M. L' Abée-Lund, Lotte P. S. Arnesen and Marina Aspholm assisted in drafting the manuscript. All authors have read and approved the final version of the manuscript.

### Conflict of interest statement

The authors declare that the research was conducted in the absence of any commercial or financial relationships that could be construed as a potential conflict of interest.
