# Supplementary material for: Commensal E. coli Stx2 lysogens produce high levels of phages after spontaneous prophage induction
Source: Front Cell Infect Microbiol. 2015 Feb 3;5:5. doi: 10.3389/fcimb.2015.00005 (PMC4315091; doi:10.3389/fcimb.2015.00005)
Supplement: Supplementary file 1 [file Table1.DOCX]

**Supplementary material**

**Table 1.** *E. coli* strains used in the study

| ***E. coli* strains** | **Serotype** | **Relevant characteristics** | **Reference** |
| --- | --- | --- | --- |
|  |  |  |  |
| NIPH-11060424 | O103:H25 | Norwegian EHEC outbreak, 2006 | (L'Abée-Lund et al., 2012; Schimmer et al*.*, 2008) |
| C600  DH5  NVH-1034 | *E. coli* K-12  *E. coli* K-12  non-O103 | laboratory strain  laboratory strain  human commensal | (Appleyard, 1954)  (Hanahan, 1985)  This study |
| NVH-1036 | non-O103 | human commensal | This study |
| NVH-1037 | non-O103 | human commensal | This study |
| NVH-1038 | non-O103 | human commensal | This study |
| NVH-1039 | non-O103 | human commensal | This study |
| NVH-1040 | non-O103 | human commensal | This study |
| NVH-1041 | non-O103 | human commensal | This study |
| NVH-1042 | non-O103 | human commensal | This study |
| NVH-1064 | non-O103 | human commensal | This study |
| NVH-1065 | non-O103 | human commensal | This study |
| NVH-1066 | non-O103 | human commensal | This study |
| NVH-1067 | non-O103 | human commensal | This study |
| NVH-1068 | non-O103 | human commensal | This study |
| NVH-1069 | non-O103 | human commensal | This study |
| NVH-1070 | non-O103 | human commensal | This study |
| NVH-1071 | non-O103 | human commensal | This study |
| NVH-1072 | non-O103 | human commensal | This study |
| NVH-1073 | non-O103 | human commensal | This study |
| NVH-1074 | non-O103 | human commensal | This study |
| NVH-1075 | non-O103 | human commensal | This study |
| NVH-1076 | non-O103 | human commensal | This study |
| NVH-1077 | non-O103 | human commensal | This study |
| NVH-1078 | non-O103 | human commensal | This study |
| NVH-1079 | non-O103 | human commensal | This study |
| NVH-1080 | non-O103 | human commensal | This study |
| NVH-1081 | non-O103 | human commensal | This study |
| NVH-1083 | non-O103 | human commensal | This study |
| NVH-1084 | non-O103 | human commensal | This study |
| NVH-1085 | non-O103 | human commensal | This study |
| NVH-1086 | non-O103 | human commensal | This study |
| NVH-1087 | non-O103 | human commensal | This study |
| NVH-1088 | non-O103 | human commensal | This study |
| NVH-1089 | non-O103 | human commensal | This study |
| NVH-1090 | non-O103 | human commensal | This study |
| NVH-1091 | non-O103 | human commensal | This study |
| NVH-1092 | non-O103 | human commensal | This study |
| NVH-1093 | non-O103 | human commensal | This study |
| NVH-1094 | non-O103 | human commensal | This study |
| NVH-37  NVH-40  NVH-317  NVH-660  NVH-661  NVH-662  NVH-676  NVH-677  NVH-679  NVH-680  NVH-681  NVH-682  NVH-729  NVH-735  NVH-806  NVH-807  NVH-808  NVH-809  NVH-810  NVH-811  NVH-827  NVH-829  NVH-832  NVH-834  NVH-835  NVH-909  NVH-927  NVH-1018  NVH-1019 | O103  O103:H7  O103  O103:H2  O103:H25  O103:H2  O103:H2  O103:H2  O103:H2  O103:H8  O103:H2  O103  O103:H2  O103:H25  O103  O103:H2  O103:H2  O103:H2  O103:H2  O103:H2  O103:H25  O103:H25  O103:H25  O103:H25  O103:H25  O103:H21  O103  O103:H25  O103:H25 | human clinical isolate  faeces, sheep  human clinical isolate  human clinical isolate  human clinical isolate  human clinical isolate  human clinical isolate  human clinical isolate  isolate from cattle  isolate from cattle  food isolate  CB5500  CB8086  CB8956  CB9286  CB9469  meat isolate  meat isolate  meat isolate  meat isolate  isolate from sheep  isolate from sheep  CDC 08-201  CDC 08-202 | This study  This study  This study  This study  This study  This study  This study  This study  This study  This study  This study  This study  This study  This study  This study  BfR, Germany  BfR, Germany  BfR, Germany  BfR, Germany  BfR, Germany  This study  This study  This study  This study  This study  This study  This study  CDC, Atlanta GA  CDC, Atlanta GA |
