# Supplementary material for: Commensal E. coli Stx2 lysogens produce high levels of phages after spontaneous prophage induction
Source: Front Cell Infect Microbiol. 2015 Feb 3;5:5. doi: 10.3389/fcimb.2015.00005 (PMC4315091; doi:10.3389/fcimb.2015.00005)
Supplement: Supplementary file 2 [file Table2.DOCX]

**Supplementary material**

**Table 2.** *E. coli* strains susceptible to lytic infection by three different phage filtrates in the spot assay. The number of tested strains is shown parentheses.

| *E. coli* strain | **Phage filtrate** | | |
| --- | --- | --- | --- |
|  | EHEC NIPH-11060424  original outbreak strain | EHEC NIPH-11060424  carrying ɸ734Cm | *E. coli* C600  carrying ɸ734Cm |
| **Strain serotype** |  | | |
| **O103** | 22 (30) | 22 (30) | 0 (30) |
| **Commensals**  **Non-O103/O157** | 0 (38) | 0 (38) | 0 (38) |
